# Kappa opioid receptor control of motivated behavior revisited

**DOI:** 10.1038/s41386-025-02226-9

**Published:** 2025-10-13

**Authors:** Zahra Z. Farahbakhsh, Cody A. Siciliano

**Affiliations:** Department of Pharmacology, Vanderbilt Brain Institute, Vanderbilt Center for Addiction Research, Vanderbilt University, Nashville, TN USA

**Keywords:** Motivation, Reward, Translational research

## Abstract

Since its discovery, the kappa opioid receptor (KOR) has held the curiosity of basic and clinical researchers across disciplines. Recent advances in technologies for measuring and manipulating KOR activity have poised the field for breakthrough discoveries. As paradigms shift, it is paramount that lessons from the foundational literature are synthesized and passed forward to inform future studies. At the same time, the promise of leveraging the KOR system to improve treatment of neuropsychiatric disorders has thus far failed to materialize, highlighting the need for critical evaluation of whether current frameworks have proven reliable and generalizable. To this end, we review the physiology and pharmacology of KORs and dynorphins and their role in motivated behavior from their discovery through present day. We focus on distinct epochs within the literature and, in parallel sections, present (A) a timeline of major findings leading to the frameworks on which current research is based, and (B) critically revisit the empirical support for specific aspects of these conclusions. In particular, we highlight discrepancies which subvert the canon that KORs’ essential function is to encode negative affective states. By synthesizing the foundational literature and calling into question long-standing theories of KOR function, we hope to highlight the ideas that should remain foundational moving forward and identify areas that may benefit from reconceptualization.

## Introduction: why psychopharmacological mechanisms matter

**Fig. 1:**
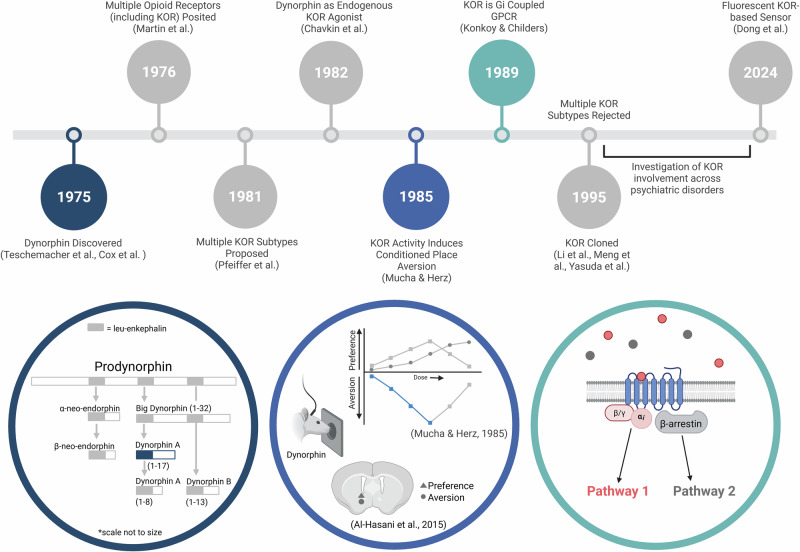
Complexities of the KOR system. Timeline showing the years of influential findings in the KOR field. Colored times indicate findings that are now understood to be far more complex than previously thought, with the original finding in color and our broader understanding in gray. Left. Dynorphin A was isolated, but we now know of a wide range of dynorphin fragments with a wide range of affinities at both KORs as well as the mu and delta opioid receptors (adapted from Margolis et al. 2023 [326]). Center. Original reports indicated that KOR agonism induced conditioned place aversion, however, different agonists and doses failed to produce aversion, rats have been shown to self-administer dynorphin, and different subregions of the nucleus accumbens produce aversion and preference. Right. Found to signal through G_i/o_ pathways, it was broadly understood that KOR activation inhibits the cell. More recent work has demonstrated that the KOR signals through a wide range of cascades and different agonists can be biased towards different downstream pathways.

In 1976, the kappa opioid receptor (KOR) was discovered and given its name based on its unique pharmacological properties [1]. In the decades since KOR was discovered, the peptide, dynorphin, was deemed its endogenous ligand, downstream signaling cascades have been characterized, its genetic sequence identified, and its functional role across diverse behavioral domains has been investigated (Fig. 1). Non-selective, multi-target drugs with actions at KORs have proven safe and effective in treating diverse ailments including: migraine [2], nociceptive [3] and neuropathic pain [4], substance use disorders [5, 6], and irritable bowel syndrome [7, 8]. Through preclinical work, it has been suggested that selective KOR agonists and antagonists hold improved therapeutic potential over non-selective ligands as well as potential for treating additional pathologies including anxiety and depression. However, clinical application of selective KOR ligands in the treatment of these disorders has proven difficult [9], and many clinical studies have found minimal, or no, effect of selective KOR modulation on primary outcome measures [10, 11].

Why have interventions supported by so much preclinical work failed in clinical trials? Fully grasping the psychopharmacological mechanisms of the KOR system is paramount if the potential of KOR ligands as therapeutics is to be realized. Importantly, while the predicted beneficial effects of KOR ligands in treating neuropsychiatric disorders have not translated (NCT02641028 [10], NCT05550532 [12], NCT06514742 [12], NCT05455684 [12]), their effects on neural activity and highly specific behaviors tested in controlled environments do appear to translate as expected (NCT02218736) [13, 14]. The disorders and symptoms targeted in the failed clinical trials were largely based on results, and associated psychopharmacological models, from preclinical testing in animals. Models are necessary to provide a framework for predicting drug effects outside of the specific scenarios in which they are tested; however, even within preclinical work, the predictive validity of our current models is not clear and often not explicitly evaluated. Instead, frameworks implicating highly specific underlying psychological mechanisms are often used to explain the results of studies with a single outcome measure, despite the fact that any single outcome measure can be explained by a plethora of latent variables [15]. Similar to the clinical outcomes, the preclinical literature quickly becomes murky when considering studies with intersectional assessments and more complex behaviors, which often have found opposite effects of KOR-targeted pharmacology than would be predicted by current theories. Together, this suggests that our models for conceptualizing KOR control of motivated behaviors may lack predictive validity within and across species, as opposed to a failure of animal-to-human translation per se.

Ultimately, the predictive power of preclinical KOR research for developing new therapeutics will remain low without better uncovering the mechanisms by which KORs modulate behaviors related to psychiatric illnesses [16]. Understanding the psychopharmacological mechanisms by which KORs modulate adaptive and maladaptive behavior would allow for accurate prediction of *who* would benefit from the treatments, *when* intervention would have the greatest efficacy, and *how* the pharmacology (e.g., sensitivity, selectivity, bias) should be optimized. With the goal of assessing the strengths and weaknesses of the standing theories of KOR function, we review the basic properties of KORs (Section 1), dynorphins (Section 2), and their influence on motivated behavior (Section 3) from their discovery through present day (Fig. 1). Each of these three topics are divided into subsections covering (A) a timeline of major findings which led to the currently accepted views and (B) dissenting and unresolved findings from the same eras and studies. By revisiting historical literature from the canonical perspective and questioning some of the conclusions from these works in parallel, we aim to provide a thought- and experiment-provoking primer for newcomers and aficionados alike.

## Physiology and neuropharmacology of kappa opioid receptors - 1A

When an opioid binding site was first discovered in the central nervous system in 1973, it was purported that opioid agents acted through a single opioid receptor [17–19]. The possibility of multiple opioid receptors had been suggested prior but direct experimental evidence was lacking [20, 21]. The theory of multiple opioid receptors was brought to the forefront when, in 1976, Martin and colleagues observed that the administration of the opioid compounds morphine, ketocyclazocine, and SKF-10,047 resulted in three distinct syndromes. It was proposed that each compound was an agonist for separate opioid receptors that were thus denominated the mu (µ, morphine), kappa (κ, ketocyclazocine), and sigma (σ, SKF-10,047) opioid receptors in reference to the name of their respective agonist [1]. Further support for the multiple opioid receptor hypothesis came from the lack of cross-tolerance and differential withdrawal symptoms observed between various opioid agonists [1]. While it quickly became accepted that there was indeed more than one endogenous opioid receptor, the exact number and members of the receptor family has been a topic in flux and controversy for much of the field’s history. Revisions to the three receptor theory began quickly: less than a year later, evidence of another receptor subtype with no response to morphine but inhibition by the pan-opioid receptor antagonist naloxone led to the characterization of the delta (δ) opioid receptor, named after the *vas deferens*, the tissue preparation in which it was discovered [22, 23]. Then, it became apparent that the sigma receptor was not of the opioid family due to the failure of naloxone to block the activity of sigma agonists [24], and its limited homology with mu and kappa opioid receptors which was revealed when the receptors were cloned [25]. An epsilon (ε) opioid receptor was also hypothesized but remains putative as no genetic origin has yet been identified [26–28]. Eventually, continued work resulted in a preponderance of evidence in support of the three-receptor theory – mu, kappa, and delta – and it became widely accepted [29] (though some now include zeta [30] and nociceptin [31] receptors in the opioid receptor family).

Characterization of the opioid receptors was rapidly attempted revealing that the KOR is a G_i/o_ coupled G protein coupled receptor (GPCR) expressed pre- and post-synaptically [32] through much of the brain, including the nucleus accumbens, ventral tegmental area, raphe nuclei, spinal cord, hypothalamus, amygdala proper, extended amygdala, and claustrum [33]. Activation of the KOR results in cellular hyperpolarization and, in neurons, decreased neurotransmitter release through a reduction in calcium and increase in potassium conductance [34–36]. Through these mechanisms, KORs are thought to regulate the release of many signaling molecules including: γ-aminobutyric acid (GABA), glutamate, serotonin, dopamine, and corticotropin-releasing factor. As an inhibitory GPCR, agonist activity at KORs results in activation of the Gα_i_ subunit and its dissociation from the Gβγ subunit. Early studies demonstrated that the Gα_i_ subunit inhibits adenylyl cyclase and thus cAMP production [37] and the Gβγ subunit results in cellular inhibition via two primary mechanisms: activation of G-protein coupled inwardly rectifying potassium channels (GIRK) and downregulation of voltage-dependent calcium channels [38–40]. Moreover, activation of the KOR and subsequent Gβγ binding inhibits voltage-gated ion channels which results in decreased calcium influx and thus reduced neurotransmitter release [41–43].

Through these mechanisms, KOR agonism in vivo results in inhibition of multiple circuits and neurotransmitter systems. Much of the systems-level in vivo literature has focused on the mesolimbic dopamine system as a readout of KOR activity, as KORs are densely expressed in accumbal dopamine terminals [44], where they act to reduce tonic dopamine levels [45–48], decrease probability and magnitude of phasic release events [49–51], and decrease terminal excitability [52–54]. KOR’s inhibitory influence over dopamine release is a major site of drug and alcohol-induced plasticity, thought to play a critical role in driving addiction [52–60]. In turn, the clinical literature has built on these conclusions, and interactions with the dopamine system are currently posited as a primary mediator of KORs’ therapeutic potential for treating neuropsychiatric disorders.

As the complex nature of KORs’ influence on cellular activity became apparent, so did the noncanonical pharmacological profiles of many of its ligands. Efforts to investigate the location and function of KOR binding sites in the central and peripheral nervous systems using synthetic ligands showed a multiplicity of binding sites that appeared to vary dramatically across putative KOR agonists or displayed differential sensitivity to antagonists. In the early 1980s, these incongruent results led to the hypothesis that there were at least two KOR subtypes, referred to as kappa_1_ and kappa_2_ [61–64] and, briefly, kappa_3_ and kappa_4_, as well as kappa_1a_ and kappa_1b_ subtypes were believed to exist [65–67]. The KOR subtype theory steadily lost traction throughout the 1990s upon successful cloning of the KOR [68–72] when only one gene encoding the receptor was found (*OPRK1*; ENSG00000082556 in human). The primary KOR agonist which was thought to be kappa_2_ preferring, bremazocine, did in fact show significant residual binding in *OPRK1*-knockout mice; however, experiments in triple opioid receptor knockouts (µ^–/–^, κ^–/–^, δ^–/–^) revealed a complete lack of bremazocine binding in whole-brain homogenates, demonstrating that the effects ascribed to kappa_2_ were ostensibly a mixture of kappa agonism and moderate off-target actions at mu and delta [73, 74]. The discovery of the nociceptin receptor (*ORL1*) in 1994 is thought to account for many of the effects ascribed to kappa_3_ [31, 75, 76]. Yet the understanding that KORs are encoded by a single gene reopened the question of which of the many profiles reported were purely KOR mediated and how one receptor system could be involved in such a multiplicity of interactions and disparate profiles.

### Kappa opioid receptors revisited - 1B

The signaling cascade downstream of KOR activation may provide answers to the question of KOR multiplicity, though a definitive model has yet to be established. In addition to G protein cascades, β-arrestins also interact with the GPCR upon ligand binding and are canonically thought to be involved in receptor internalization and desensitization [77]. However, it has been found that β-arrestins also induce p38 mitogen-activated protein kinase signaling, which then activates diverse cellular events (e.g., transcription, channel phosphorylation) [78–80]. Originally thought to be static on/off processes downstream of GPCR activation, more recent work has demonstrated that different ligands are able to recruit these various cascades to different extents [81]—i.e., ligands can be *biased* towards different pathways [82–84].

For the KOR in particular, it has been suggested that G protein and β-arrestin signaling are responsible for different components of the effects of KOR activation, and many KOR ligands are biased towards one or the other, thus modulating separate behaviors despite acting on the same receptor **(**Fig. 1). For example, p38 mitogen-activated protein kinase signaling, a β-arrestin dependent signaling cascade, has been shown to be functionally important to the motivational properties of KOR activation. KOR activity in ventral tegmental area dopaminergic neurons is sufficient to induce an aversive response to a KOR agonist but this effect is completely negated when β-arrestin recruitment is blocked or p38α MAPK is knocked out, despite G protein activity remaining intact [80]. This signaling cascade has also been shown to be necessary for stress-induced immobility (but not KOR-mediated analgesia), a behavior endogenously controlled by KOR activity [85]. Evidence that each signaling cascade downstream of the KOR is responsible for specific behavioral repertoires suggests that the bias inherent in many KOR compounds may, at least in part, explain the varying outcomes observed with KOR activation. More recently, there has been movement to capitalize on the bias of specific agonists to have one effect (e.g., analgesia) while limiting another (e.g., dysphoria) to improve clinical utility of KOR-targeted compounds [86].

Another developing area of research that offers a putative explanation for the multiplicity of effects seen across KOR ligands is work demonstrating functional hetero-oligomerization of KORs with various other membrane proteins [87]. Among the most studied are delta opioid receptor-KOR oligomers [88, 89]. Cells co-expressing KOR and delta opioid receptors show a dramatic reduction in binding of multiple selective ligands, including the KOR ligands U69,593 and NorBNI, compared to cells expressing KOR or delta receptors in isolation [89]. Other KOR-containing oligomer complexes that are thought to form include KOR homo-oligomers [90, 91] as well as hetero-oligomers with the mu opioid receptor [91, 92], neurotensin receptor [93], and dopamine transporter [94]. It is thought that formation of these complexes is state-dependent, given that agonists reduce the density of KOR-delta oligomers [89, 95], and their occurrence can also be sexually dimorphic, as is the case with KOR-mu opioid receptor oligomers which preferentially form in females [92]. Recent work suggests that opioid receptors rapidly toggle between states, forming transient dimers which last just a few hundred milliseconds, concomitant with differential coupling to downstream effectors [96]. However, a method for assessing the formation of oligomer complexes in vivo remains a long-standing challenge, and it is not yet clear when and where these complexes form in intact tissue.

Regarding KORs influence on neuronal activity and neurotransmitter release in vivo, the literature is more mixed than the conclusions above indicate, even for straightforward outcome measures such as dopamine overflow measured via microdialysis. Several of the studies reporting KOR-induced inhibition of striatal dopamine also noted biphasic actions whereby *higher* doses had little to no effect on dopamine levels [47, 48] while several others have failed to show any effect of KOR agonists [56, 97] or found agonist-induced increases in dopamine levels [98]. The inverse dose-dependent effects may potentially be explained by off-target interactions at other receptor systems acting in the opposite direction; nonetheless, this warrants further investigation given that the dosages are commonly used and interpreted as KOR selective. Another intriguing and currently unaddressed finding is that the behavioral effects of KOR agonism were not entirely ablated in *OPRK1*-knockout animals, even when using the KOR selective agonist U50,488H [99]. These findings indicate that there may yet be a second KOR subtype or, more likely, that the commonly used dose range for in vivo application of KOR ligands is less selective than currently thought. This possibility is consistent with the fact that most kappa ligands are highly potent at other receptors even when fold selectivity is high **(**Table 1). For example, NorBNI displays more than 100-fold selectivity for KOR over mu and delta, making it a highly selective antagonist which has therefore been often used as validation that a particular effect is KOR-mediated; however, because it is also extraordinarily potent (K_i_ at KOR is ~260 picomolar), concentrations as low as 50 nanomolar will likely be non-selective, and by the mid-nanomolar range it is likely to be acting as a full pan-opioid antagonist [100]. Combined with the fact that the central pharmacokinetics of many KOR ligands are not well characterized, this creates a situation in which, despite using selective ligands, there is a high likelihood of off-target actions when considering the literature *en masse*, particularly for in vivo assays. The need for greater consideration of both fold-selectivity and absolute potency of KOR ligands when selecting dosage and interpreting prior results is a theme that is raised throughout this review.

**Table 1:** Relative affinities of opioidergic compounds.

Compound	K_i_ at KORs (nM)	κ / μ / δ (K_i_ Ratio)	Canonically considered a	Also known as
**Apadoline** [298, 299]	0.55	1 / 104 / 20.7	Selective KOR agonist	RP 60180
**Aticaprant** [300, 301]	0.8	1 / 30 / 194	Selective KOR antagonist	LY-2456302, JNJ-67953964, CERC-501, Opra Kappa
**β-chlornaltrexamine** [302]	2.5	1.25 / 1 / 32	Pan-opioid irreversible antagonist	β-CNA
**Bremazocine** [69, 303]	0.11–0.67	1 / 1.3 / 4.3	Non-selective KOR agonist	
**BU09059** [304]	1.72	1 / 15/ 616	Selective KOR antagonist	
**Butorphanol** [305, 306]	2.5	4 / 1 / 29	Non-selective partial KOR agonist (57% efficacy)	
**Cyclazocine** [307]	0.18	1 / 2 / 6	Non-selective KOR agonist	Win-20,740, ARC II-C-3
**E-2078** [69, 308]	1.5–1.9	1 / 2.3 / 14.2	Selective KOR agonist	Dynorphin A(1–8) ethylamide
**Enadoline** [309]	0.11	1 / 905 / 9418	Selective KOR agonist	CI-977
**Ethylketocyclazocine** [69, 307]	0.62-1.6	1/ 1.25 / 5.5	KOR agonist	MR 2266
**HS665** [310–312]	0.49	1 / 1106/ > 20,000	Partial KOR agonist (90% efficacy)	
**HS666** [310–312]	5.9	1 / 140 / > 1700	Partial KOR agonist (53% efficacy)	
**Ketocyclazocine** [307]	1	1 / 3 / 20	Prototypical KOR agonist	Ketazocine
**Morphine** [69, 303, 313]	47-1900	26 / 1 / 88	Prototypical mu agonist	
**MR 2034** [69, 254]	1.3	1.3 / 1 / 2.4	Pan-opioid agonist	
**Nalfurafine** [314, 315]	3.5	1 / 15 / 343	Selective biased KOR agonist (G protein biased)	Remitch, TRK-820, AC-820, MT-9938
**Nalmefene** [316, 317]	0.08	1 / 29 / 1920	Non-selective partial KOR agonist (25% efficacy)	Revex
**Naloxone** [158]	2.3	2 / 1 / 18	Prototypical pan-opioid antagonist	Narcan, Evzio
**Naltrexone** [69, 158, 318]	1.1-3.9	4 / 1 / 149	Non-selective pan-opioid antagonist	EN-1639A, Revia, Depade, Vivitrol
**Niravoline**	Unknown	Unknown	Reported as a selective KOR agonist [319]	RU 51599
**Nor-Binaltorphimine** [69, 100]	0.26	1 / 169 / 153	Long-acting selective KOR antagonist	NorBNI
**Noribogaine** [320–322]	720	1 / 2 / 20	Non-selective biased partial KOR agonist (75% efficacy, G protein biased)	
**Salvinorin A** [323]	4.3–16	1 / > 625 / > 625	Selective KOR agonist	
**SKF-10,047** [303]	4.7	1.5 / 1 / 5	Prototypical sigma agonist	N-allyl-N-normetazocine
**Spiradoline** [324]	8.6	1 / 29 / 1093	Selective KOR agonist	U-62,066E
**Tifluadom** [325]	0.78	1 / 2.5 / 196	Selective KOR agonist	
**U69,593** [69, 153]	0.3–2.0	1 / 3817 / > 30,000	Selective KOR agonist	
**U50,488** [69, 303]	1.9-15	1 / 55 / 1400	Selective KOR agonist	U50,488H, U50,488E

Pharmacodynamic properties of KOR ligands and related compounds mentioned throughout this review.

## Physiology and neuropharmacology of dynorphins - 2A

Close in time to the discovery of multiple opioid receptors came the first isolation of endogenous substances in the brain with morphine-like characteristics [101]. Among these was “dynorphin”, isolated from porcine pituitary in 1975 and named from the Greek *dynamis* (power) in reference to its incredible potency [102–104]. In a matter of a few years, a slew of related peptides were isolated and it became clear that dynorphins composed a large family of neuropeptides that shared their first few amino acids. This group of peptides includes: dynorphin-A(1–8), dynorphin-A(1–17), dynorphin-B, α-neo-endorphin, β-neo-endorphin, leumorphin, and big-dynorphin [105–110], though several were isolated by different groups concurrently and have been known by different names (for a detailed review of opioid peptide nomenclature see [111]). Big-dynorphin (1-32) is the largest of these dynorphin peptides and is composed of dynorphin-A and dynorphin-B linked by two amino acids which are thought to serve as a cleavage site [112–114]. The first five residues in these peptides are thought to be crucial to conferring opioid activity; fragments without these residues have been found and are thought to be biologically inactive [115].

In one of the first characterizations of dynorphin signaling, it was found to be highly potent in the guinea pig ileum bioassay of opioid activity, and displayed decreased sensitivity to inhibition by naloxone compared to mu and delta ligands. Intriguingly, dynorphin displayed similar potency to ethylketocyclazocine, a synthetic KOR agonist, leading to the hypothesis that dynorphins are the endogenous KOR ligand [103]. Further evidence of dynorphin being a KOR ligand came through competitive inhibition assays with KOR agonists [116], different assay preparations that were thought to have varying levels of each of the opioid receptors [117], radioligand binding assays [118], and cross tolerance tests that demonstrated that pretreatment with mu or delta agonists had no effect on dynorphin potency, but KOR agonists did [119]. To demonstrate dynorphin’s preferential affinity for KORs and validate that the opioid receptors are indeed physically distinct, *β*-chlornaltrexamine, an irreversible, covalently-binding, pan-opioid antagonist, was used in a receptor protection assay. Pretreatment of guinea pig ileum with dynorphin peptide protected the ethylketocyclazocine-sensitive binding sites from inactivation by *β*-chlornaltrexamine [120], demonstrating that the synthetic ligand ethylketocyclazocine and endogenous peptide dynorphin shared the same high affinity target (i.e., KORs).

Eventually, it became clear that all of the neuropeptide fragments within the dynorphin family are derived from the precursor prodynorphin [121]. Preprodynorphin mRNA is translated to the precursor preprodynorphin peptide which is then cleaved by signal peptidases to yield prodynorphin, the precursor from which all dynorphin fragments are derived (for review of dynorphin biosynthesis see [122]). Prodynorphin is processed, at least in part, by prohormone convertase 2 at several cleavage sites [123]. It was originally thought that prodynorphin was cleaved prior to transport to synaptic sites, however, evidence that prodynorphin and dynorphin fragments are colocalized in some axon terminals suggests that local processing at or near synaptic release sites may occur [124]. Dynorphin is packaged within large dense core vesicles, lipid vesicles that are larger (typically 80–200 nm diameter) and electron-denser (i.e., appearing dark on an electron micrograph) than their canonical vesicle counterparts [125]. Dynorphins are found within both axon terminals and dendrites [126, 127] and, like KORs, are widely distributed throughout the central nervous system [122, 128, 129]. Dynorphin is also often co-packaged in vesicles with other neuropeptides such as hypocretin [130] and vasopressin [131]. Interestingly, dynorphin fragments have different potency for the KOR and the proposed order (from most to least) is dynorphin-A (1–17), then big-dynorphin, dynorphin B-29, α-neo-endorphin, and dynorphin-B; and finally, dynorphin-A (1–8) and β-neo-endorphin [132]. Beyond potency however, the field has yet to reach a consensus on whether the various fragments are distinct in terms of their cellular actions or phenomenological functions (Fig. 1).

Regarding their phenomenological function as a family, dynorphins have largely been conceptualized in juxtaposition with endorphins, which are associated with positive emotional states. Dynorphin levels, as measured by immunoreactivity, have been shown to increase in various brain regions in response to stress hormones as well as stressful experiences (e.g. immobilization, forced swim) [133–135], and dynorphin knockout prevents avoidance of stressor-paired stimuli [136] as well as changes induced by repeated forced-swim [137]. Increased dynorphin tissue content, dynorphin release, and expression of preprodynorphin mRNA are associated with acute and chronic exposure to variety of substances of abuse, including ethanol, cocaine, nicotine, opiates, and psychostimulants [138–145]. Congruent with a role in stress, dynorphin levels are also heightened in various brain regions during periods of withdrawal from ethanol [52, 146–148] and morphine [149]. The findings implicating dynorphin in stress- and substance-induced behavioral adaptations have led to the prevailing model that dynorphins counter positive emotional drives, acting as a stressor and an aversive signal through their actions at KORs.

### Dynorphins revisited - 2B

Though dynorphins do act on KORs [150], it is important to note that they also are highly potent ligands at other receptors. Dynorphin fragments can be cleaved into leu-enkephalin, an endogenous opioid with high affinity for mu and delta opioid receptors [151] and even the KOR-active dynorphin fragments have been shown to have nanomolar affinities (ranging by fragment) for the mu and delta opioid receptors [152–154]. To put these concentrations to scale, dynorphins' affinity for mu and delta receptors is higher than dopamine’s affinity for dopamine receptors, for example [155, 156]. Additionally, KORs also respond to the other endogenous opioids, with the mu opioid agonists (endorphins) and the delta opioid agonists (enkephalins) having nanomolar and micromolar affinity, respectively, for KORs [153, 157, 158]. Thus, though each opioid receptor may have a preferential endogenous agonist, it is highly likely that there is promiscuous signaling between the opioid receptors and opioid peptides [154, 159, 160]. Though there is considerable indirect evidence which is congruent with this possibility (e.g., difficulty recapitulating the behavioral effects of dynorphin with selective KOR agonists [161]), it largely has not been incorporated into the field. Accordingly, dynorphins are almost ubiquitously discussed as the endogenous ligand for KORs, which connotates exclusivity over other systems. Going forward, it will be critical to determine what concentration dynorphins reach in vivo, and in what compartments, to allow for clear interpretation of its potency at KORs and beyond.

In addition to possible effects across opioid receptors, dynorphins have been shown to have non-opioid mediated effects, including substantial evidence that multiple dynorphin fragments, including dynorphin A and big dynorphin, directly modulate NMDA receptors [162–169]. Interestingly, there is evidence that opioid-active dynorphins can be cleaved into non-opioid binding fragments that are able to induce physiological effects even in the absence of KOR binding [170, 171]. Finally, a highly intriguing and rapidly developing area of research has highlighted the apparent membrane permeabilizing effects of dynorphins. Multiple groups have shown that big dynorphin and dynorphin A accumulate in cell membranes and intracellular compartments by opening channel-like giant pores in plasma membranes (estimated to be up to 2.7 nm in diameter) [172–175]. These properties have been proposed as a putative mode of signal transduction, but whether this is a toxic effect or one of adaptive biological function remains unclear. In sum, there are many mechanisms through which they can signal and much work is yet to be done to dissociate the various contributions of each of these systems.

Though dynorphins are typically conceptualized as stressors (i.e., mediating the subjective experience of stress), there is considerable evidence that their relationship with stressful stimuli is far from one-to-one. Multiple studies have highlighted brain-region and stimulus-type specific effects of stress on dynorphin levels. For example, cold swim stress and cold stress decreased hypothalamic dynorphin levels while restraint, tail pinch, and starvation stress had no effect [176, 177]. Along these same lines, preprodynorphin mRNA expression in the nucleus accumbens has been shown to be increased by acute social defeat stress but decreased by chronic social defeat stress, and dynorphin tissue content is unchanged following acute or chronic social isolation stress [178, 179]. Though there are certainly many examples in the literature of stressful stimuli increasing dynorphin (see Section 2A), the fact that acute administration of reinforcing drugs and alcohol is associated with increased dynorphin levels [180–183] runs counter to a causal relationship with stress and aversion. Likewise, administration of dynorphin or the KOR agonist enadoline in humans does not alter circulating cortisol [184, 185] while the KOR antagonist acticaprant acutely increased cortisol levels [11, 186]. In contrast, some preclinical studies have found an increase in corticosterone or corticotrophin releasing hormone levels after KOR activation [187–190] and a reduction in corticosterone levels in dynorphin knock-outs [191] (but see [192, 193]). Together, these results implicate dynorphins in responsivity to stress but suggest that conceptualization of dynorphin as a inherent stressor may not allow for predictions which generalize outside of certain specific experimental conditions.

## Kappa opioid receptor control of motivated behavior - 3A

KORs’ value as a target for pharmacological intervention is in part due to its unique motivational and behavioral properties that differentiate it from the mu and delta opioid receptor systems. For example, KOR preferring compounds are of particular interest as analgesics given that, like mu agonists, they have analgesic effects, but, unlike mu agonists, have limited abuse potential and are not associated with a severe withdrawal syndrome [194–196]. Despite some aversive properties of KOR agonists, agonist-induced conditioned place aversion is absent in models of chronic pain suggesting that these compounds may still serve as viable analgesics [197]. It has also been widely posited that upregulation of the KOR system leads to motivational disturbances and contributes to the negative affective state underlying many psychiatric disorders such as depression and substance use disorders [45, 53, 55, 58, 138–144, 147, 198–203].

Initial preclinical investigations of opioid receptor control of motivation utilized intravenous self-injection paradigms as a measure of reinforcing or addicting properties of drugs [204] and found that animals will self-administer non-specific and mu-specific opioid receptor agonists [205]. KOR ligands, however, were found to have a distinct profile which was consistent across agonists and species [196, 206–208]. Unlike non-specific opioid agonists, early studies showed that animals self-administer selective KOR agonists to a limited degree, or not at all [196, 209]. However, this assay cannot distinguish between a neutral versus aversive stimulus: both would result in a failure to maintain responding (but see [210]). The development of the conditioned place preference assay, wherein the subject has concurrent access to a previously drug-paired and control context [211–213], addressed this by providing a bidirectional measure of preference. Congruent with the self-administration studies, non-specific and mu-specific opioid receptor activation induce robust place preference [214–216]. Additionally, a number of studies found that KOR agonists (e.g., U50,488 and E-2078) induce conditioned place aversion and concluded that KOR activation is aversive [215, 217]. This was further supported by the finding that KOR agonists could also induce conditioned taste aversion when animals had a choice between an agonist- or control-paired tastant [215]. Further investigations revealed that the dosage required to induce place aversion was far lower when delivered via intracerebroventricular infusion compared to subcutaneous injection, and concluded that the aversive effects are centrally mediated [217].

Congruent with these psychopharmacological conclusions and the types of experience-dependent plasticity that have been observed in this system (see Sections 1A and 2A), KOR antagonists have shown preclinical potential in the treatment of depression- and addiction-like behaviors. As would be hypothesized from evidence that dynorphin levels increase after stress, in rodent models of depression, KOR antagonists have been shown to reduce forced swim stress immobility and learned helplessness [133, 218–220]. However, despite evidence of KOR and dynorphin upregulation after substance use, the causal role of the system’s increased signaling in addiction was initially difficult to demonstrate. In contrast to expectations, many studies demonstrated that KOR activation (not antagonism) decreased self-administration or conditioned preference for substances of abuse [221–225]. However, later work showed that administration of KOR antagonists has experience-dependent effects, whereby they reduce ethanol and cocaine self-administration only in subjects with prolonged, chronic exposure to the substance [57, 58, 226–229]. After induction of dependence, the effect of KOR signaling is seemingly especially important in withdrawal and stress states; KOR antagonism selectively attenuates ethanol and nicotine deprivation effects, and blocks stress-induced reinstatement and potentiation of substance use in animal models [137, 198, 230–235].

Regarding the acute effects of KOR activation, the findings from animal studies have been largely mirrored by behavioral pharmacology experiments in humans. In humans, intravenous infusion of a KOR agonist, MR 2034, is psychotomimetic and induces anxiety [236]. Likewise, the selective agonist enadoline (CI-977) in humans induced visual distortions, depersonalization/dissociation, and dose-dependent increases in ratings of “bad effects” of the drug [185, 237]. Similar effects including confusion/abnormal thinking, auditory and/or visual hallucinations, and distortion of personality/self have been reported with multiple other KOR agonists including ketocyclazocine [238], niravoline (RU 51599) [239], and salvinorin A [240].

Together, these findings have led to consensus that (1) pharmacological activation of KORs is aversive, (2) these effects are centrally mediated, and (3) the endogenous function of the KOR and dynorphin system is to encode aversion, negative valence, and motivation driven by avoidance of negative stimuli. In turn, these conclusions motivated testing of KOR ligands for mood disorders and have heavily influenced which disorders have been targeted, as well as the implementation strategies utilized.

### Kappa opioid receptor control of motivated behavior revisited - 3B

KORs and dynorphins’ putative role as an ‘anti-reward’ or ‘negative reinforcement’ system has provided a framework to explain observations of KOR-mediated effects on behaviors associated with stress and substance use. However, the literature discussed above contains several findings regarding the motivational properties of KOR activation that conflict with current interpretations and warrant further consideration (Fig. 1). For example, though animals will not self-administer U50,488 [196], other KOR agonists are self-administered, including ethylketocyclazocine [205, 241, 242]. Additionally, ethylketocyclazocine and tifluadom appear to induce place preference while the aversive effect of U50,488 (the more selective agonist) is bimodal, with *higher* doses failing to demonstrate conditioned avoidance [215, 217]. There is some speculation that this may be due to off-target effects on the mu opioid receptor, but this claim has not been sufficiently investigated. Additionally, Mucha & Herz (1985) report that mu agonists, compounds widely considered to have euphoric properties, also induce conditioned taste aversion in their paradigm, thereby undermining the conclusion that the presence of taste aversion with KOR agonism is necessarily due to a dysphoric effect [215].

Further complicating the picture, a number of studies have concluded that dynorphin itself is reinforcing, even when delivered to the central nervous system. Indeed, rodents will self-administer dynorphin microinfusions into the hippocampus, demonstrating that it can function as a positive reinforcer [243, 244]. Some studies have also found that systemic administration of KOR agonists and intracerebroventricular administration of dynorphin can induce conditioned place preference [245, 246]. Likewise, Salvinorin A — a recreationally used KOR agonist with hallucinogenic effects—has been shown to be reinforcing in rats and zebrafish, an effect which was blocked by the KOR antagonist NorBNI [98, 247]. More recently, we directly tested a central tenet of the aversive processing theory, which states that inhibition of KORs decreases motivation driven by negative reinforcement contingencies (i.e., the removal of aversive stimuli); counter to this prediction, we found that KOR antagonism augmented learning rate across both positive and negative reinforcement contingencies. KOR blockade did not modulate anxiety-like behaviors, or innate responses to positive (sucrose consumption) or negative (shock reactivity) stimuli, again suggesting KORs act on a valence-independent domain [248].

Another factor that may contribute to multifaceted behavioral effects of KOR agonism is the specific localization of KORs that are recruited by pharmacological manipulations. For example, while early work showed that microinjections of KOR agonists into the ventral tegmental area and nucleus accumbens induced place aversion [249], more recent investigations show differential effects of KOR activation across the rostral/caudal and dorsal/ventral axes of the nucleus accumbens shell. Agonist microinjections and optogenetic stimulation of preprodynorphin-lineage cells in the rostrodorsal or rostral nucleus accumbens shell resulted in appetitive responses, such as place preference and decreased anxiety-like behaviors [250–252]. In each of these studies, activation outside of this rostrodorsal zone resulted in behaviors indicating aversion, in line with previous reports of the system. These findings demonstrate complexity in the KOR system; beyond mediating only aversion and dysphoria as has long been accepted, the system appears to drive preference in specific subregions.

It is also widely accepted that the aversive properties of KOR activation are centrally mediated, though several groups have argued the opposite. For example, systemic U50,488 only induced place aversion at low doses, and vagotomized (severed vagus nerve) rats showed conditioned place preference [253]. The authors concluded that the low dose locally activated peripheral KORs and drove aversion, while higher doses acted on the central nervous system and counteracted the effects of peripheral activation. Of note, reports on both sides, those claiming central versus peripheral mediation of aversive effects, relied on blood brain barrier permeable compounds (U50,488 and E2078) to draw their conclusions [217, 253]; it will be critical for future work to use compounds modified to restrict their activity to either the central or peripheral compartment to determine the veracity of these claims.

Many of the inconsistencies in animal studies noted above are recapitulated in studies of the behavioral pharmacology of KOR ligands in human subjects, often within the same studies that are cited to support the claim that KORs’ essential function is to encode aversive states. One of the publications most widely cited to support the claim that KOR activation is aversive, Pfeiffer et al. 1986, actually reports no effect on overall mood [236]. While this study did report that high doses of MR 2034 (3.8 µg/kg, i.v.) were psychotomimetic and anxiogenic compared to subjects receiving MR 2034 plus naloxone, no significance vs pre-drug baseline was noted and a placebo condition was not included [236]. Critically, MR 2034 is not KOR-selective or preferring, as it displays picomolar affinity for mu receptors and low-nanomolar affinity for delta opioid and sigma-1 receptors [254, 255] (Table 1). Another study widely cited as evidence of KORs being synonymic with aversion, Walsh et al. 2001 [237] reported psychotomimetic and dysphoric effects of 160 µg / 70 kg of intramuscular enadoline but this dosage was only tested in two volunteers as a pilot; within the study design, all doses tested (up to 80 µg / 70 kg i.m.) were well-tolerated with no major adverse events. Among the human studies discussed in Section 3A that did show direct dysphoric effects of KOR agonists, these actions were only observed at high doses, though many other self-reported and physiological changes were noted with lower doses [185, 237–239, 256, 257], and several studies have reported psychotomimetic/hallucinogenic effects concomitant with positive effects on mood/liking [240, 258–263]. When dysphoric components of KOR agonism have been observed it is with doses equal to or greater than those which also produce psychotomimetic effects [185, 237, 238]; given that in healthy subjects hallucinations and psychotic events are rare, it is reasonable to assume that endogenous activation of KORs under most conditions is not sufficient to induce psychotic, and by extension dysphoric, effects. In agreement with this interpretation, both short- and long-acting KOR antagonists do not acutely induce euphoria or elevations in mood in healthy subjects or substance use disorder populations [10, 11, 264], suggesting that ongoing endogenous activation of KORs is not sufficient to influence mood. Also contrary to the idea that upregulated KOR activity drives the negative affective component of withdrawal, dynorphin has been shown to be well-tolerated and to reduce opiate withdrawal symptoms in both human and preclinical models [265–267].

When extrapolating from the results of exogenous pharmacological stimulation to infer the endogenous function of the KOR system, it is critical to acknowledge that many, perhaps all, neuromodulatory systems can produce aversive and psychotomimetic effects when pushed to an extreme. Indeed, dopamine [268–270], serotonin [271], adrenergic [272], and even mu opioid [273–275] receptors can produce dysphoria, confusion, and hallucinations when stimulated to supraphysiological levels. Thus, the high incidence of reports associating KOR agonists with psychotomimetic effects may simply reflect the fact that most KOR agonists are extremely potent, typically with affinities in the high picomolar to low nanomolar range (Table 1). Further highlighting the dose-dependency of KOR agonist-mediated dysphoria, when given at low doses, multiple studies, including two double-blind placebo controlled studies, have reported that salvinorin-A (10–20 µg / kg, smoked or inhaled) is not only well tolerated, but has actions comparable to classical serotonergic psychedelics, and induced effects such as increased laughter and movement [240, 260–262].

## Predictions for clinical applications

The findings discussed in Sections 3A and 3B unequivocally demonstrate that KOR activation *can* be aversive but also cast considerable doubt on whether KORs *encode aversion*. The distinction is not trivial: as explained in the introduction of this review, if the translational value of KOR therapeutics is to advance, we need to be able to predict the impact of KOR modulation of behavior in untested scenarios. To do so, we must gain an understanding of the essential function that the system endogenously subserves, as opposed to fitting *post hoc* explanations. Importantly, the framework of conceptualizing KORs as subserving aversion and negative affect has formed the basis of the last several decades of efforts in developing KOR therapeutics with central actions. Thus far, these efforts have been unsuccessful, with just one centrally active KOR selective ligand, nalfurafine, being approved for clinical use. Even in this successful case, the outcomes were not predicted by the standing model: nalfurafine was originally developed as an analgesic and after failing in clinical trials due to adverse events related to sedation, not dysphoria, it was repurposed at a lower dose to successfully treat uremic pruritus [276–278]. Though nalfurafine’s efficacy in treating uremic pruritus and its dose-limiting effects on sedation were not predicted by the standing models, it was selected for testing based on preclinical data demonstrating potent anti-itch effects [277, 279–281], lending further credence to the notion raised throughout this review that animal-to-human translation is not to blame for the lack of success of KOR ligands in treating neuropsychiatric indications. Here, we outline an alternative conceptualization and some of the readily testable predictions from this model regarding clinically relevant outcomes of KOR modulation.

**Fig. 2:**
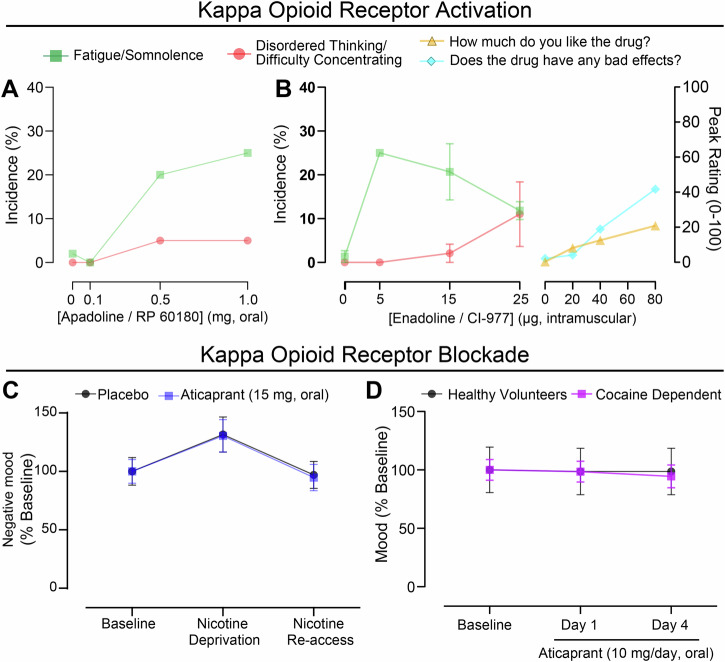
Low dose KOR agonists induce somnolence without dysphoria and KOR antagonists do not acutely modulate mood. **A** Dose-response function for apadoline/RP 60180 (K_i_ at κ / μ / δ = 0.55 nM / 57.1 nM / 11.4 nM) [298, 299] demonstrating increased fatigue/somnolence at doses which are not psychotomimetic. No dysphoric effects were reported at any of the dosages tested. The 0 dose represents a placebo condition. Data are reproduced from Lötsch et al., 1997 [298]. **B** Dose-response function for enadoline/CI-977 (K_i_ at κ / μ / δ = 0.11 nM / 99 nM / 1036 nM) [309] demonstrating (left) increased fatigue/somnolence induced by doses which are not psychotomimetic and (right) that subjective ratings of drug liking and bad effects do not appreciably diverge until doses of 80 µg, a 16-fold greater dose than the peak of the dose-effect function for fatigue/somnolence. Note that the dose-effect for fatigue/somnolence is on the descending limb where effects in the psychotomimetic domain begin, suggesting recruitment of a second receptor or effector system. The 0 dose represents a placebo condition for both graphs. Left: data were combined from Reece et al. 1994 [185], Pande et al. 1996a [256], and Pande et al. 1996b [257] and represent the mean ± SEM calculated across the three studies for this review. Right: data were adapted from Walsh et al. 2001 [237]. **C** Blockade of KORs with 15 mg of oral aticaprant (K_i_ at κ / μ / δ = 0.8 nM / 24 nM / 155 nM, see Table 1 for drug aliases) did not alter negative mood scores in cigarette smokers under baseline conditions, following 18 h of abstinence from smoking, or following smoking at the end of the abstinence period. Data adapted from Jones et al. 2019 [10] (baseline normalized for comparison, data represent the mean ± SEM). **D** Blockade of KORs with 10 mg of oral aticaprant taken daily for 4 days did not alter mood in healthy subjects or cocaine dependent subjects during early abstinence. Baseline represents a pre-treatment assessment one day prior to treatment day 1. Data adapted from Reed et al. 2018 [11] (baseline normalized for comparison, data represent the mean ± SEM). Numerical data from Walsh et al. 2001 were extracted using PlotDigitizer; all other replotted data were originally reported in tabular format and thus were manually entered into GraphPad Prism (V10) to create these visualizations.

Recently, results from human and animal studies have found that KOR antagonists increase learning rate, without altering hedonic responses to reinforcers, and attenuate stimulus habituation [13, 14, 248]. We have proposed modulation of novelty processing as a model for the global action of KORs, a framework that can also explain why activation of this system can produce aversive (i.e., neophobic) as well as appetitive (i.e., neophilic) reactions depending on dosage and context [248]. Consistent with this hypothesis, effects of KOR agonism on fatigue, sedation, and/or somnolence were observed at lower doses than psychotomimetic or dysphoric actions in the human behavioral pharmacology studies summarized above (Fig. 2A, B) [185, 237, 239]. Further, blockade of KORs via the short-acting antagonist aticaprant acutely increases circulating cortisol in healthy controls as well as cocaine dependent subjects [11, 186]; antagonist-induced increases in cortisol is opposite of the view that KOR activation is a stressor, but is again congruent with novelty processing, as circulating cortisol is well-documented as a primary physiological driver of novelty seeking in humans and animals [282–288]. While it remains to be determined if this framework will prove useful in predicting the outcomes of KOR manipulations in untested conditions, we posit that there is a clear need for reconceptualization and that testing predictions based on broad axioms is the most efficient route towards determining which are worth further refinement.

The dose-dependent actions of KOR effects in humans and animals provide critical insights for therapeutic targeting. At low doses, KOR agonists consistently produce sedation and fatigue without the dysphoric or psychotomimetic effects observed at higher concentrations. Enadoline for example, a highly selective KOR agonist displaying greater than 900-fold and 9000-fold selectivity over mu and delta opioid receptors, produces marked increases in sedation/fatigue at doses as low as 5 µg (~70 ng/kg assuming a 70 kg average participant weight) (Fig. 2A, B). Notably, self-reports of disordered thinking only began at doses 5 times greater, and self-reported bad effects of the drug only diverged from drug liking at doses 16 times greater. This suggests a wide therapeutic window for conditions where controlled sedation or reduced arousal is beneficial, such as certain sleep disorders, bipolar mania, and attention deficit hyperactivity disorder. These applications would leverage the more predictable, lower-dose effects of KOR activation rather than attempting to harness analgesic properties that emerge from higher doses associated with significant side effects. Following the same line of reasoning, KOR antagonists may be useful for conditions that display poor responsivity to stimulants but may benefit from moderate potentiation of arousal, such as myalgic encephalomyelitis/chronic fatigue syndrome.

Regarding the clinical utility of selective KOR antagonists for mood disorders, though both the negative affect/dysphoria model and the novelty processing model predict that KOR antagonism could reduce depression and excessive drug use, the two frameworks diverge regarding implementation strategy. In the case of addiction, if KOR antagonists are increasing novelty responsivity and reinforcement, the mechanism for reduced drug taking would be via augmented reinforcing value of alternative, new reinforcers relative to the highly familiar (i.e., non-novel) substance and the engrained drug taking behaviors observed after chronic use. This would be consistent with the preclinical literature in which KOR antagonists decrease intake following extended drug exposure, while administration during initiation of drug use (when the stimuli are still novel) or in subjects with limited access (when stimuli have undergone less habituation) does not [226–228, 289, 290]. On the other hand, a negative reinforcement model would suggest that the mechanism for reduced intake is via decreased negative affect during periods of abstinence. However, administration of aticaprant (see Table 1 for aliases) in humans showed no effect on mood in cigarette smokers or cocaine-dependent individuals, even during acute withdrawal **(**Fig. 2C, D) [10, 11]. Based on a novelty processing framework, we hypothesize that KOR antagonist treatment would be most effective (1) after the acute withdrawal phase when initial abstinence has already been achieved or when withdrawal symptoms are managed by a concurrent treatment, and (2) in conjunction with behavioral therapy that emphasizes alternative reinforcers, such as differential reinforcement of alternative behavior therapy [291–293]. This framework also predicts that KOR antagonists would be least effective in scenarios where alternative reinforcement and novel experiences are limited, such as in-patient and structured laboratory studies which comprise most of the testing that has been conducted thus far for substance use disorders.

Regarding the use of KOR antagonists for the treatment of major depressive disorder, the two models again diverge in the most likely implementation to produce efficacious results. According to the negative affect framework, KOR antagonists would hold the most therapeutic potential for the treatment of individuals with low or depressed mood, a DSM-V symptom of depression [294]. Yet, low mood is not necessary or sufficient for a diagnosis of major depressive disorder; another symptom observed in many individuals with depression is anhedonia, or the loss of interest or pleasure in activities. Within the novelty processing framework, we predict that KOR antagonism may be efficacious for anhedonia specifically, as it would increase the inherent motivational quality of novel reinforcers/activities. Interestingly, this hypothesis is consistent with recent clinical outcomes. In a fast-fail trial of aticaprant (see Table 1 for aliases) for the treatment of anhedonia, it was found that KOR antagonism reduced anhedonia as measured by ventral striatal activation to reward-predicting cues, reward learning, and self-report of the symptom (notably without reducing overall depression levels) [13, 14]. Further supporting selective actions on anhedonia specifically, KOR availability is highly correlated with anhedonia severity in patients with schizophrenia (without associations with psychosis) [295]. Similar to substance use disorders, we predict that successful outcomes would be most likely if treatment were administered in conjunction with behavioral therapy that emphasizes establishing novel activities/reinforcers.

## Conclusions

Through clever, intersectional application of a library of pharmacological compounds and a variety of ex vivo preparations, the KOR and dynorphins, were discovered. Under certain parameters, KOR activation appears to drive aversion and negative affect, but a closer dissection of historical literature uncovers numerous discrepancies and more recent literature shows evidence that the system may be involved in positive valence or, more likely, altogether valence-independent processes. With breakthroughs in KOR-targeted pharmacology and the recent advent of paradigm-shifting tools to precisely monitor and modulate KOR activity and dynorphin signaling, our ability to interrogate these systems has been vastly expanded [296, 297]. As the field works to fill long-standing gaps in our understanding of the KOR system with these new tools, it is essential that we heed the extant literature and explicitly evaluate standing models as they are carried forward, such that our frameworks are bounded by data, not assumptions.
